# Surgical site infection after trochanteric and subtrochanteric fractures: a single centre retrospective analysis

**DOI:** 10.1038/s41598-024-51180-x

**Published:** 2024-01-05

**Authors:** Thomas Sator, Harald Binder, Stephan Payr, Lorenz Pichler, Stephan Frenzel, Stefan Hajdu, Elisabeth Presterl, Thomas Manfred Tiefenboeck

**Affiliations:** 1 Department of Orthopedics and Trauma-Surgery, Division of Trauma-Surgery, Medical University of Vienna, Währinger Gürtel 18-20, 1090 Vienna, Austria; 2 Department of Infection Control and Hospital Epidemiology, Medical University of Vienna, Währinger Gürtel 18-20, 1090 Vienna, Austria

**Keywords:** Fracture repair, Geriatrics, Clinical microbiology, Medical research, Risk factors

## Abstract

Surgical site infection (SSI) following osteosynthesis of trochanteric or subtrochanteric fractures is a rare but serious complication with incidence rate ranging from 1 to 3%. SSIs are associated with higher mortality and increased length of hospital stay resulting in higher healthcare costs and loss of life quality. In this retrospective analysis all patients with SSI following osteosynthesis of trochanteric or subtrochanteric fractures at the Department of Trauma Surgery were identified. We included all surgical procedures performed from 1992 to 2018, using data from electronic health records and SSI-Trauma-Registry. The aim was to describe epidemiological data, as well as to identify parameters correlating with the occurrence of SSI and mortality. Of 2753 patients, 53 (1.9%) developed SSI. Longer operative time was demonstrated among patients with SSI (P = 0.008). Mortality during the first postoperative year was significantly higher in the SSI group (32.1% vs. 19.1%; P = 0.018), with detection of *methicillin-sensitive* (MSSA) and *methicillin-resistant Staphylococcus aureus* (MRSA; HR 4.13, CI 95% 1.24–13.80; P = 0.021) or *Enterococcus *spp. (HR 5.58, CI 95% 1.67–18.65; P = 0.005) being independent risk factors. Male sex (HR 2.25, 95% CI 1.86–2.73; P < 0.001) and higher mean age (HR 1.05, 95% CI 1.04–1.06; P < 0.001) were found to be predictors for 1-year mortality in non-infected patients. SSI rate was low with 1.9% and longer duration of surgery was associated with infection. Patients with SSI had a higher 1-year mortality, with detection of MSSA, MRSA and enterococci significantly increasing the risk of dying. Male sex and higher age were risk factors for one-year mortality in patients without SSI.

## Introduction

Proximal femoral fractures are among the most common fractures to be treated by surgery and represent a major health care problem in developed countries. In Europe alone, more than 600,000 hip fractures occur each year^1^.

With the elderly population increasing every year, the number of hip fractures will grow proportionally as will the associated burden on the health care system^2^. The annual incidence of such fractures will more than double in near future, with the number of hip fractures expected to exceed 6 million worldwide by 2050^3,4^.

Trochanteric fractures account for more than 40% of all hip fractures and can be treated with intra- and extramedullary implants. Fractures of the subtrochanteric region occur less frequently^5–8^. For these fractures, intramedullary nailing is currently considered the gold standard^8,9^.

Surgical site infection (SSI) is among the most common hospital-acquired infections. However, the likelihood of postoperative wound infection occurring varies depending on patient and type of surgery^10,11^. SSI following osteosynthesis of trochanteric and subtrochanteric fractures occur in approximately 1–3% of patients^12–16^. These infections lead to prolonged hospital stays, higher treatment costs, reduction in quality of life and increased mortality^12,14–17^.

There are ample studies describing SSI after hip fracture surgery. However, most of these studies did not differentiate between femoral neck fractures and trochanteric or subtrochanteric fractures, even though the surgical treatment of these differs substantially^12,14–16^.

Still, there are no defined guidelines on how to proceed in the event of an infection following osteosynthesis of trochanteric and subtrochanteric fractures. Although recent studies have discussed possible treatment methods for fracture-associated infections, there are still no implant related recommendations^18^.

The aim of this study was to describe the incidence of SSI and the most common bacteria and antibiotics involved in these fractures. Furthermore, potential risk factors for SSI were to be assessed and mortality rates at given follow-up times to be investigated.

## Methods

Using the SSI trauma database, we identified all patients who underwent osteosynthesis for trochanteric or subtrochanteric fractures at the Department of Trauma Surgery at the Vienna General Hospital, an 1800-bed academic hospital (Medical University of Vienna). Data was collected within the period of January 1992 to January 2018. Cases with surgery time of less than 10 or more than 180 min were excluded as were patients below 18 years of age at time of surgery and those treated with hip arthroplasty. Surveillance of SSI was performed according to the protocol HAISSI of the European Centres of Disease Prevention and Control (ECDC).

**Figure 1:**
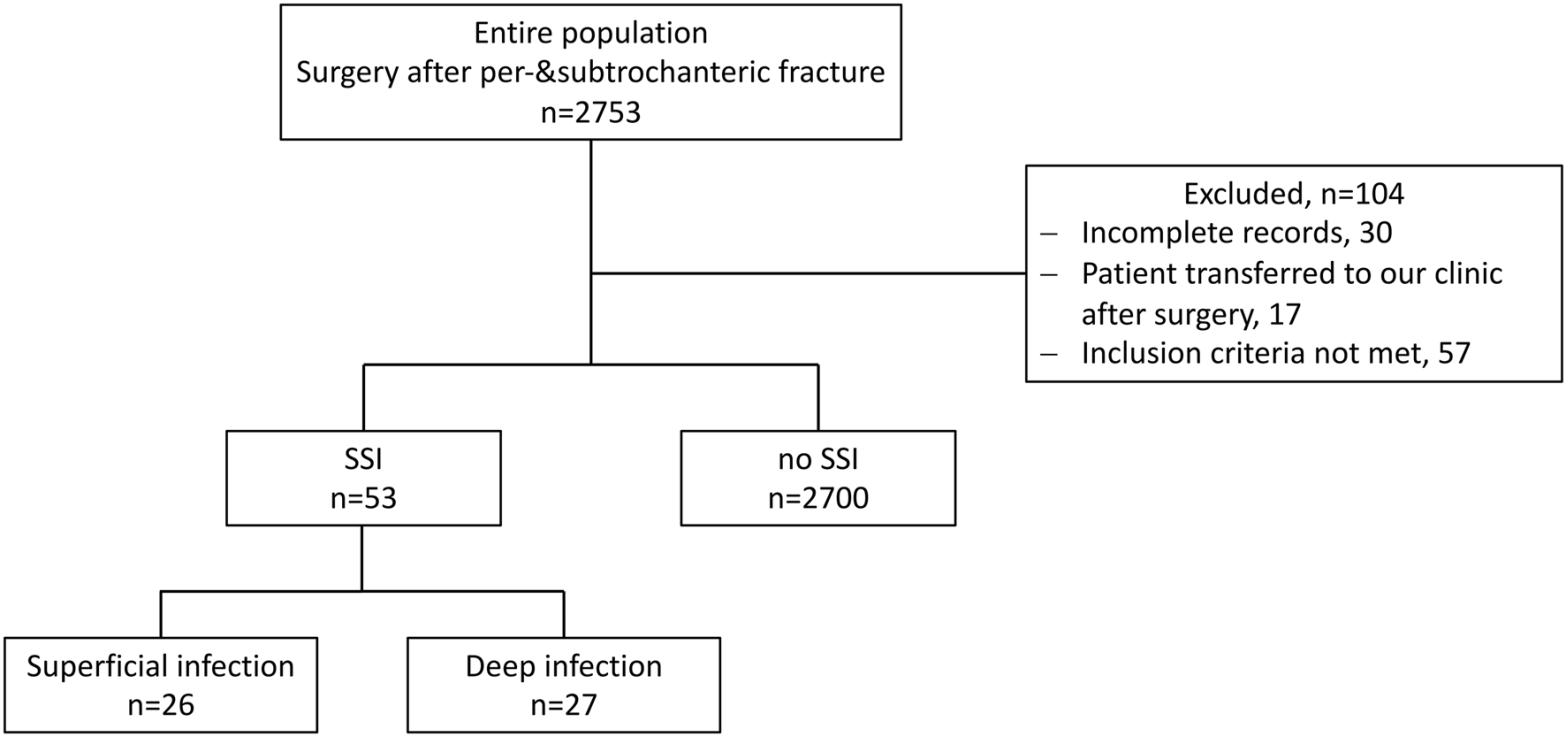
Flowchart of patient inclusion and overview of infections occurred. *SSI *Surgical site infection.

Using these criteria, a total of 2753 cases were identified which were subsequently investigated for SSI (Fig. 1). Only cases that fulfilled the criteria of the ECDCs HAI-Net SSI Protocol^19^ were included. Information regarding SSI occurrence, pathogen detection, antibiotic therapy and pre-existing conditions were obtained from the patient’s charts stored in the electronic patient management system of the Vienna General Hospital.

Data collected included demographic variables (sex and patient age at injury), duration of surgery, implants, complications, and revision surgery. Charlson Comorbidity Index (CCI) was used to assess comorbid conditions. All surgical procedures were performed in laminar flow operation theatres at the Department of Trauma Surgery. Preoperative antibiotic prophylaxis was administered in every case, with the current standard being the i.v. application of 1.5 g cefuroxime approximately 30 min before start. Prior to 2008, 1 g of amoxicillin/clavulanic acid was administered preoperatively. In case of allergy to penicillin or cephalosporins, clindamycin was used.

Mortality was determined after 30 days, 3 months, and 1 year, with time measured from the start of documentation to last follow up. Descriptive data (mean, +−SD) were reported for the entire study population.

### Statistical analysis

Categorical data was tested with either Chi-square test or Fishers exact test. Differences in continuous variables were compared by Student’s t-test if they corresponded to normal distribution. Mann–Whitney-U-Test was performed where results were not normally distributed. For survival analyses we used Kaplan–Meier curves, log-rank test, and Cox regression to evaluate differences between patients. Risk factors for mortality, which showed statistically significant influence in univariate analysis were further analysed in a multivariate Cox regression model to determine independent predictors. Schoenfeld residuals and log-minus-log survival plots were performed for all covariates to verify the proportional hazards assumption. In all analyses a P-value < 0.05 was considered to be statistically significant. Data obtained was analysed using Microsoft Excel®, SPSS® Version 23 (IBM Corp., Armonk, N.Y., USA) and GraphPad Prism® Software (GraphPad Software Inc., San Diego, CA, USA).

### Ethics approval and consent to participate

The retrospective analysis of data was carried out in compliance with the principles outlined in the Declaration of Helsinki and approved by the Ethics Committee of the Medical University of Vienna (Ethics Commission Code: 1215/2015). In accordance with the retrospective nature of the study, requirement for written informed patient consent was waived, which was approved by the Ethics Committee of the Medical University of Vienna.

## Results

A total of 2753 patients [2031 (73.8%) female, 722 (26.2%) male] who underwent surgery for trochanteric or subtrochanteric fractures were screened for SSI (Table 1).

**Table 1:** Demographics and risk factors for SSI following fracture fixation after trochanteric and subtrochanteric fracture.

	SSI	No SSI	Total	P-value
Entire population (%)	53 (1.9%)	2700 (98.1%)	2753 (100%)	–
Sex				*0.553*^*†*^
Female, n (%)	41 (1.5%)	1991 (72.3%)	2032 (73.8%)	*–*
Male, n (%)	12 (0.4%)	709 (25.9%)	721 (26.2%)	*–*
Mean age, years (range, SD)	77.7 (44–97, 14.2)	80.2 (18–104, 12.6)	80.2 (18–104, 12.6)	0.177*
Mean duration of surgery, minutes (range, SD)	74.2 (20–145, 32.3)	61.7 (13–180, 28.9)	61.9 (13–180, 29.0)	0.008*
Mean length of hospital stay, days (range, SD)	57.2 (10–103, 24.6)	14.1 (1–93, 8.3)	16.0 (1–103, 13.0)	< 0.001*
Mortality				
After 30 days, n (%)	1 (1.9%)	140 (5.2%)	141 (5.1%)	*0.523*^*‡*^
After 3 months/90 days	7 (13.2%)	283 (10.5%)	290 (10.5%)	*0.522*^*‡*^
After 1 year, n (%)	17 (32.1%)	517 (19.1%)	534 (19.4%)	***0.018***^*†*^

^†^Chi-square test.

^‡^Fisher exact test.

*Mann–Whitney-U test.

Significant values are in bold and italic.

53 patients developed SSI demonstrating an overall incidence rate of 1.9%. Of these 53 patients, 41 (1.5%) were female and 12 (0.4%) were male. Mean duration of surgery for patients without infection was 61.7 (+/− 28.9 SD) minutes, compared to 74.2 (+/− 32.3 SD) minutes for those with SSI—a difference which proved to be of statistical significance (P = 0.008). Among patients with SSI, the mean age was 77.7 (+/− 14.2 SD) years and therefore lower than in the non-infection group with 80.2 (+/− 12.6 SD) years. However, no significant difference could be shown.

Concerning surgical techniques applied intramedullary implants were used in most cases n = 42 (79.2%), with dynamic hip screwing less frequently carried out n = 11 (20.8%). The mean length of hospital stay was 14 days in patients without postoperative wound infection and 57 days in those with SSI (P < 0.001).

### Microbiology and type of infection

Out of the 53 (1.9%) patients with SSI, 26 (0.9%) had superficial infection and 27 (1.0%) had infections classified as deep. Implant removal was performed in 8 cases (15.1%), while in 45 patients (84.9%) the implant stayed in situ.

A total of 63 different pathogens were identified in microbiological swabs and tissue samples derived from the infection sites in 47 patients. Collection of the samples was always performed in the operating room. In 6 patients, microbiological results were missing. The most common pathogen found was *methicillin-sensitive Staphylococcus aureus* (MSSA; n = 15, 24%), followed by *coagulase-negative staphylococci other than Staphylococcus epidermidis* (n = 11, 17%) and *Enterococcus *spp. (n = 8, 13%). *Methicillin-resistant Staphylococcus aureus* (MRSA) accounted for 8% (n = 5) of all SSIs and was only detected in patients with deep infection. Polymicrobial infection was found in 13 (28%) cases and most common where deep infections occurred (n = 10, 21%). Table 2 depicts the pathogens divided into superficial and deep infections.

**Table 2:** Pathogens identified from microbial cultures, divided into superficial and deep infections.

Pathogens	Superficial incisional SSI	Deep incisional SSI
*Staphylococcus aureus* (all), n	8	12
Methicillin-sensitive *S. aureus*	8	7
Methicillin-resistant *S. aureus*	0	5
*Enterococcus* spp., n	2	6
Coagulase-negative Staphylococci, n	6	5
*Staphylococcus epidermidis*, n	2	4
*Escherichia coli*, n	1	2
*Pseudomonas aeruginosa*, n	1	2
*Proteus mirabilis*, n	1	1
*Corynebacterium* spp., n	0	2
Group A ss-haemolytic streptococci, n	1	0
Enterobacter cloacae, n	0	1
No growth, n	5	1
Total	27	36
Polymicrobial, n	3	10

### Antibiotic therapy

After SSI onset, antibiotic therapy was established in 47 patients (88.7%). Combination therapy of > 1 antibiotic was found in 13 cases (27.7%). Penicillin’s accounted for 33% (n = 30) of therapies, followed by clindamycin (n = 14, 15%), glycopeptide antibiotics (n = 10, 11%), cephalosporins (n = 10, 11%) and fluoroquinolones (n = 9, 10%). The most common combination therapies were levofloxacin plus teicoplanin (n = 2, 15.4%) and ampicillin/sulbactam plus rifampicin (n = 2, 15.4%). Table 3 lists all antibiotics used with average dosage and length of administration.

**Table 3:** Antibiotics administered during inpatient stay, with mean dose and duration of administration.

Antibiotics	Number	Mean dose per day (mg)	Mean duration of administration (days)
Aminoglycosides	2 (2.2%)		
Gentamicin i.v	2 (2.2%)	120	3
Carbapenems	6 (6.6%)		
Imipinem i.v	3 (3.3%)	200	10
Meropenem i.v	3 (3.3%)	4666.7	19
Cephalosporines	10 (11%)		
1st Generation^a^	4 (4.4%)	3750	6
2nd Generation^b^	1 (1.1%)	4500	17
3rd Generation^c^	2 (2.2%)	6000	14
4th Generation^d^	3 (3.3%)	4000	14
Clindamycin i.v./p.o	14 (15.4%)	1714.3	13
Fluoroquinolones	9 (9.9%)		
2nd Generation^1^	3 (3.3%)	666.7	15
3rd Generation^2^	4 (4.4%)	1062.5	33
4th Generation^3^	2 (2.2%)	400	13
Fosfomycin i.v	5 (5.5%)	12,000	14
Fusidic acid i.v./p.o	2 (2.2%)	1250	27
Linezolid i.v	1 (1.1%)	1200	14
Penicillin’s	30 (33%)		
Amoxicillin/Clav. i.v	21 (23.1%)	6285.7	11
Ampicillin/Sulbactam i.v	4 (4.4%)	7350	19
Flucloxacillin i.v	2 (2.2%)	4500	17
Piperacillin/Tazobactam i.v	1 (1.1%)	13,500	26
Others^§^	2 (2.2%)	–	–
Rifampicin i.v	2 (2.2%)	600	22
Glycopeptides	10 (11%)		
Teicoplanin i.v	5 (5.5%)	1000	15
Vancomycin i.v	5 (5.5%)	1100	19
Total	91 (100%)	–	–

^a^Cefalexin p.o. (n = 1), cefamandole i.v. (n = 3).

^b^Cefuroxime i.v. (n = 1).

^c^Cefotaxime i.v. (n = 2).

^d^Cefpirome i.v. (n = 3).

^1^Ciprofloxacin i.v./p.o. (n = 2), ofloxacin p.o. (n = 1).

^2^Levofloxacin i.v. (n = 4).

^3^Moxifloxacin i.v. (n = 2).

^§^Penicillin G i.v. (n = 1), mezlocillin/oxacillin i.v. (n = 1).

### Mortality

**Figure 2:**
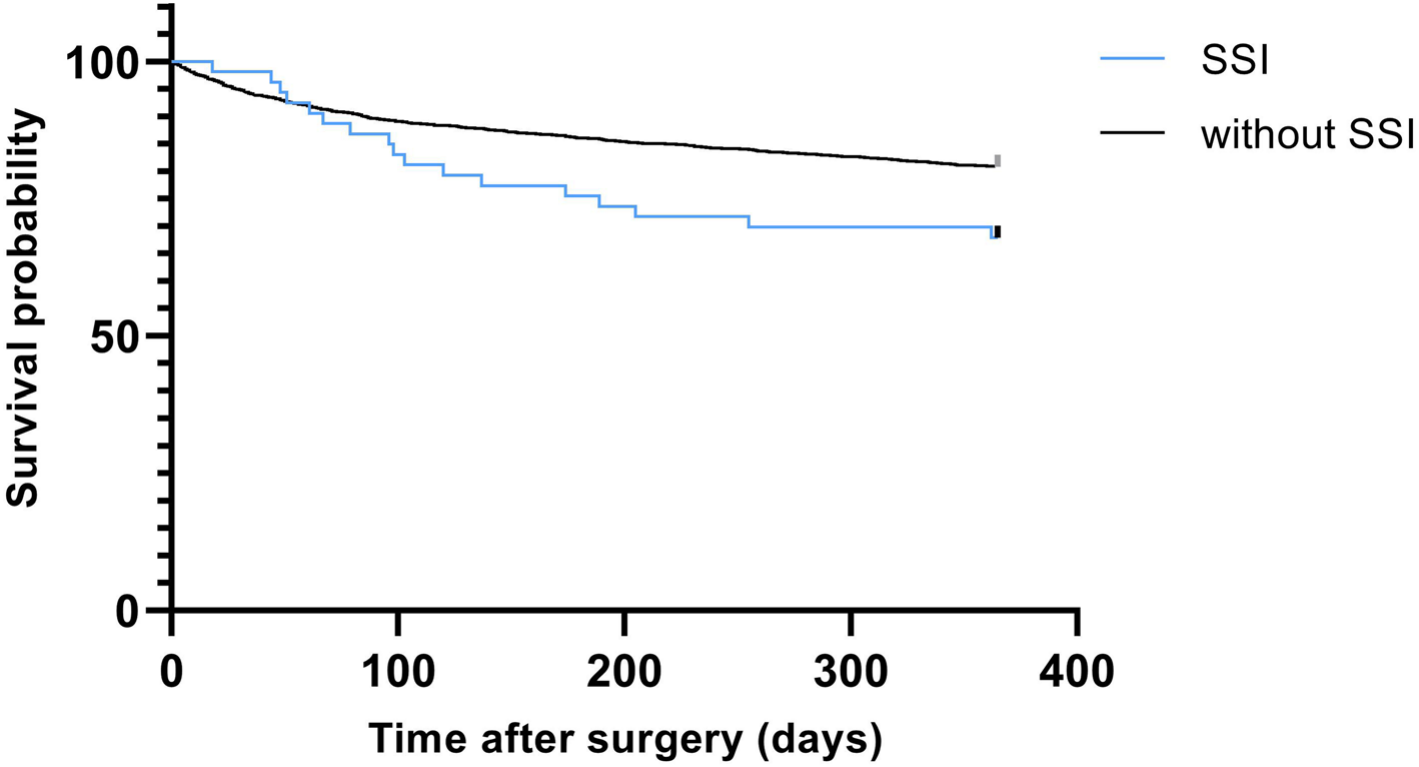
Kaplan–Meier survival curve for patients with SSI following surgery after trochanteric and subtrochanteric fracture. *SSI* Surgical site infection.

A total of 30 patients (56.6%) with SSI died during the investigated period. The 30-day mortality following SSI was 1.9%, while mortality in patients without infection was 5.2%. After six months the mortality increased significantly in the SSI group (24.5%) compared to patients without infection (14%, P = 0.030). This trend continued until the one-year follow-up with another 17 deaths occurring in the SSI group, representing a one-year mortality of 32%, compared to 517 deaths (19.3%) in the non-infection cohort—a result which also proved to be significant (P = 0.018). The Kaplan–Meier curve computed for the cumulative survival shows the increased mortality of patients with SSI becoming apparent at approximately eight weeks after surgery (Fig. 2).

### Risk factors for 1-year mortality

The second aim of this study was to analyse potential risk factors for one-year mortality in patients with and without SSI (Table 4). Adjusted multivariate analysis identified male sex (HR 2.25, 95% CI 1.86–2.73; P < 0.001) as significant predictors for 1-year mortality in the absence of SSI. Although with less impact, higher mean age (79.3 years vs. 84.3 years; P < 0.001) was also associated with increased risk for mortality after 1-year (HR 1.05, 95% CI 1.04–1.06). Duration of surgery was lower in patients who died during the 1-year follow-up period but did not prove as significant in multivariate analysis.

**Table 4:** Univariate and multivariate analysis to determine risk factors for one-year mortality in patients without SSI following osteosynthesis after trochanteric and subtrochanteric fracture.

Univariate analysis			
Risk factors	Survivors	Non-survivors	P-value
Sex			**<*0.001***
Female, n (%)	1649 (82.8%)	342 (17.2%)	–
Male, n (%)	534 (75.3%)	175 (24.7%)	–
Mean age, years (range, SD)	79.3 (18–104, 12.9)	84.3 (39–104, 10.1)	**<*0.001***
Mean duration of surgery, minutes (range, SD)	62.3 (15–180, 29.3)	59.3 (13–160, 26.7)	***0.032***

Multivariate analysis			
Risk factors		Hazard ratio (95% CI)	P-value
Male sex		2.25 (1.86–2.73)	**<*0.001***
Age		1.05 (1.04–1.06)	**<*0.001***
Duration of surgery		1.00 (0.99–1.00)	*0.384*

Significant values are in bold and italic.

Risk factors for mortality following SSI at one year postoperatively, were also described and analysed (Table 5). An ASA score of three or above turned out as a predictive factor for mortality after SSI. Regarding co-morbidities, only chronic lung disease affected mortality within a year.

**Table 5:** Univariate and multivariate analysis to determine risk factors for one-year mortality in patients with SSI following osteosynthesis after trochanteric and subtrochanteric fracture.

Univariate analysis			
Risk factors	Survivors	Non-survivors	P-value
Total	36	17	
Sex			*0.379*
Female, N (%)	29 (80.6)	12 (70.6)	*–*
Male, N (%)	7 (19.4)	5 (29.4)	*–*
Mean age, years (range, SD)	77,8 (44–97, 12.7)	79,4 (47–95, 13.3)	*0.616*
Mean duration of surgery, minutes (range, SD)	72.5 (20–140, 32.7)	77.0 (40–145, 32.6)	*0.756*
Smoking, N (%)	9 (25.0)	4 (23.5)	*0.549*
Alcohol abuse, N (%)	10 (27.8)	5 (29.4)	*0.959*
Mean BMI^1^ (range, SD)	23.5 (17.6–32.4, 3.6)	24.1 (19.7–29.3, 3.1)	*0.505*
ASA^2^ score			
ASA 1, N (%)	6 (16.7%)	1 (5.9%)	*0.347*
ASA 2, N (%)	22 (61.1%)	6 (35.3%)	*0.086*
ASA 3 to 4, N (%)	8 (22.2%)	10 (58.8%)	***0.006***
Co-morbidities			
Cardiovascular disease, N (%)	12 (33.3)	8 (47.1)	*0.352*
Chronic respiratory disease, N (%)	3 (8.3)	7 (41.2)	***0.005***
Renal failure, N (%)	3 (8.3)	1 (5.9)	*0.646*
Liver disease, N (%)	2 (5.6)	3 (17.6)	*0.166*
Neurological, N (%)	5 (13.9)	3 (17.6)	*0.723*
Dementia, N (%)	7 (19.4)	2 (11.8)	*0.531*
Diabetes, N (%)	4 (11.1)	4 (23.5)	*0.137*
Mean CCI^3^ score (range, SD)	4.4 (0–8, 1.7)	5.5 (3–12, 2.3)	*0.059*
Osteoporosis, N (%)	3 (8.3)	2 (11.8)	*0.694*
Urinary tract infection^4^, N (%)	9 (25.0)	9 (52.9)	*0.189*
Deep infection, N (%)	20 (55.6)	7 (41.2)	*0.386*
Pathogens			
Staphylococcus aureus (all), N (%)	9 (25.0)	11 (64.7)	***0.008***
MSSA, N (%)	7 (19.4)	8 (47.1)	*0.054*
MRSA, N (%)	2 (5.6)	3 (17.6)	*0.136*
*Enterococcus* spp. N (%)	3 (8.3)	5 (29.4)	***0.042***

Multivariate analysis			
Risk factors		Hazard Ratio (95% CI)	P-value
SSI with MSSA or MRSA		4.13 (1.24–13.80)	***0.021***
SSI with Enterococcus spp.		5.58 (1.67–18.65)	***0.005***
ASA 3 to 4		2.82 (0.80–9.98)	*0.108*

^1^Body Mass Index.

^2^American Society of Anaesthesiologists.

^3^Charlson Comorbidity Index.

^4^Urinary tract infection during hospital stay.

Significant values are in bold and italic.

Where MSSA or MRSA was detected as a pathogen, an increased one-year-mortality rate was shown. Infection due to enterococci also had an impact on mortality. Finding of these pathogens sustained as the only independent risk factors after multivariate analysis. SSI with MSSA or MRSA and enterococci resulted in patients having a 4.13 (95% CI 1.24–13.80, P = 0.021) and 5.58 (95% CI 1.67–18.65, P = 0.005) times higher risk of dying within the first postoperative year, respectively, than those with infection caused by other bacteria.

## Discussion

Surgical site infections following orthopaedic surgery are serious complications associated with higher mortality, longer hospital stays, and greater costs to the health care system^17,20–22^. These negative effects and the preventable nature of SSI through evidence-based interventions should emphasize the importance of SSI surveillance to ensure adequate quality of patient care^23,24^.

In our retrospective analysis, the overall SSI rate following surgical treatment of trochanteric and subtrochanteric fractures was low with 1.9%. Superficial-incisional SSI occurred in 0.9%, deep infection in 1% of cases. These results are consistent with data reported in current literature^12,14,15,25^.

Several studies described the development of SSI as multifactorial, with both patient- and procedure-related factors contributing to it^22,26,27^.

A procedure-related factor which could be confirmed to contribute towards SSI by our study is operating time. The average operating time in cases with infection was 13 min longer than in those without infection—this proved to be statistically significant (P = 0.008).

The influence of operative time on SSI has been investigated in numerous studies, some but not all found longer surgery to be associated with higher risk of infection^16,22,26,28,29^. Liu et al. demonstrated that an increased risk of SSI should be expected when operating time exceed 107 min^16^. Edwards et al. on the other hand found no association between intervention time and SSI after hip surgery^12^. An explanation for these contradictory results might be that different type of implants and therefore also different procedures were investigated. Furthermore, the correlation of operating time and surgeon experience was not considered. In our study intramedullary nailing was the therapy of choice—a procedure which comes with a rather short operating time when performed by an experienced surgeon. However, there are reports that this type of procedure is often carried out by younger surgeons as part of their residency training resulting in longer operating times. Less experienced surgeons not only take longer for procedures but are also more likely to cause contamination of surgical sites through technical mistakes possibly linking longer operating times to less experienced surgeons to more technical mistakes as an explanation of the found results^12,15^.

Advanced patient age as a potential risk factor for SSI has also been investigated in several studies^16,28,30^. In contrast, Edwards et al. and Harrison et al. did not report a significant influence of age on the development of SSI^12,15^. Our result not only confirmed Edwards et al. but even found patients without SSI to have higher mean age (80 years) than those with infection (77.7 years).

There was no significant association between gender and infection in our study, which is also in accordance with literature^15^. Patients with SSI spent an average of 57 days in hospital—43 days more than those without infection. Prolonged hospital stay following SSI is a common finding in literature and is associated with an existential increase in treatment costs^12^.

To treat SSI appropriately, early detection of the pathogen involved is essential. We found *S. aureus* (24%) to be the most common bacteria, isolated in patients with SSI. MRSA, widely discussed as a severe problem in hospital-acquired infections, accounted for only 8% of cases and was exclusively found in deep infections. Although the overall incidence of *S. aureus* was consistent with data reported in literature, MRSA was not nearly as common as described in previous studies^31–35^. However, Austria is a country with low MRSA incidence^36^. Yet, the incidence of *Enterococcus spp.* in SSI cases was approximately two times higher than reported by others^31,32,34^. Polymicrobial infection was found in 28% of all patients with SSI, which is in accordance with current literature^31,37^. Due to the retrospective study design, it is not possible to control the precision of swab and tissue collection, which is why the positive bacterial cultures for skin germs could also be due to inaccurate collection of the samples.

For all cases, antibiotic therapy was administered according to the antibiogram. Beta-lactams were used in one third of cases with high dosages of amoxicillin/clavulanic acid being the therapy of choice in most cases (23.1%). Other commonly used agents include clindamycin (n = 14, 15%), glycopeptide antibiotics (n = 10, 11%) and cephalosporins (n = 10, 11%). The frequent use of these substances can be explained by the high proportion of gram-positive cocci, which accounted for over 70% of the microbiological spectrum found. No pre-existing studies reporting on the variety of antimicrobial therapies used in SSI after osteosynthesis of trochanteric and subtrochanteric fractures was found. Zimmerli et al. however presented recommendations on pathogen-specific therapy in implant-associated infections. If the implant remains in situ and *S. aureus* is detected, combination of rifampicin with *S. aureus*-sensitive antibiotics is advised due to its beneficial biofilm activity. Pathogen-specific antibiotics without biofilm activity are recommended if the implant is removed^38^. Regarding this recommendation, therapy with rifampicin was carried out only in 2 cases included in this study. In both cases, a combination therapy with ampicillin/sulbactam was administered. The low number of this therapeutic regimen can possibly be attributed to the rapid surgical revision with appropriate debridement and insertion of carrier material for local antibiotic delivery such as gentamicin-loaded chains. In most cases, the implant remained in situ.

There are ample studies describing one-year mortality rates after SSI following surgery for hip fracture, with rates 5–10% above non-infection cases, ranging from 33 to 50%^12,14,15,25,34^. At 32% mortality the results of our study are on the lower end of the reported range but still significantly higher when compared to patients without SSI (19.3%, P = 0.018).

We hypothesize that the lower mortality for patients with SSI at our clinic might be related to the high volume of cases with these fractures treated compared to smaller hospitals resulting in a higher expertise on their management. However, mortality rates shown in our study are still high. What should be considered is that the population suffering frequently from these fractures are of old age alongside several general diseases. Mean CCI-Score in patients with SSI in our study was between 4 and 5 points correlating for a one-year mortality of 54% to 86%. Therefore, our use of mortality is a crude outcome, as patients may have died because of events unrelated to infection or relevant treatment.

When it comes to the 30-day follow-up interestingly the opposite result could be observed: A higher mortality in patients without SSI (5.2% vs. 1.9%). The reason for this could be that especially older patients with severe comorbidities died before any postoperative complication occurred like wound infection, e.g. Again, Edwards et al. came to the same results, identifying higher mortality within the first 30 days for non-infected patients and increasing after one year in those with SSI^12^.

We also described potential risk factor for one-year mortality in our study. In non-infected patients, male gender (P < 0.001) and higher patient age (P < 0.001) could be identified as independent predictors for mortality, which has been described in studies before^25,39,40^.

An ASA score of three or above and chronic lung disease proved to be significant risks for unadjusted one-year mortality among patients with SSI. Both factors, however, were not statistically significant in multivariate analysis. In contrast a study by Duckworth et al. showed that age, diabetes, and dementia were the only risk factors after hip fracture surgery. ASA score and other pre-existing conditions had no significant influence on mortality in their multivariate analysis^34^.

When it comes to pathogens, infection with MSSA, MRSA or *Enterococcus *spp. revealed as independent risk factors for one-year mortality after SSI in both univariate and multivariate analysis. SSI with these bacteria resulted in patients having a higher risk of dying within the first postoperative year than those with infection caused by other bacteria. The work of Partanen et al. accorded with this and attested the occurrence of these pathogens a higher mortality in patients with deep infection following fracture fixation^14^.

Since this work was a retrospective data analysis, study design-related limitations must be considered. Due to insufficient documentation regarding some surgical reports, as well as microbiology and antimicrobial therapy, especially in older patient data, not all cases could be fully considered. Furthermore, it should be considered that the surgical treatment was performed by surgeons with different levels of experience leading to a certain bias. Regarding the significant difference in OP duration, a selection bias must be assumed due to the small number in the SSI group.

Because of the limited number of corresponding risk factors for SSI and one-year mortality in multivariate analysis, the sample might not be representative, which is why larger numbers are recommended for further studies on infection surveillance. Despite these limitations, the results of this study provide a good overview of the postoperative infection rate and mortality after surgical treatment of trochanteric and subtrochanteric fractures at a level-I trauma centre.

## Conclusion

SSI following osteosynthesis for trochanteric and subtrochanteric fractures is a rare but serious complication. It was shown that longer duration of surgery is associated with the occurrence of SSI. Mortality in the first postoperative year was significantly higher for patients with SSI. Male sex and older age were independent risk factors for one-year mortality among patients without infection. Detection of MSSA or MRSA and enterococci showed to be independent predictors for mortality in the SSI group resulting in patients having much higher risk of dying within the first postoperative year than those with infection caused by other bacteria. Patients with these risk factors should be followed up closely to reduce the chances of problematic infection and therefore mortality.

## Data Availability

The dataset analysed in this study is available from the corresponding author on reasonable request.

## Abbreviations

SSI: Surgical Site Infection
ECDC: European Centre for Disease Prevention and Control
MSSA: Methicillin-sensitive *Staphylococcus aureus*
MRSA: Methicillin-resistant *Staphylococcus aureus*
ASA: American Society of Anaesthesiologists
BMI: Body Mass Index
CCI: Charlson Comorbidity Index
SD: Standard deviation
